# Impact of marathon performance on muscles stiffness in runners over 50 years old

**DOI:** 10.3389/fpsyg.2023.1069774

**Published:** 2023-02-24

**Authors:** Krzysztof Mackala, Dariusz Mroczek, Paweł Chmura, Marek Konefał, Damian Pawlik, Bartosz Ochman, Jan Chmura, Bartłomiej Paleczny, Rafał Seredyński, Małgorzata Wyciszkiewicz, Adrianna Nowicka-Czudak, Wojciech Łopusiewicz, Dorota Adamiec, Szczepan Wiecha, Piotr Ponikowski, Beata Ponikowska

**Affiliations:** ^1^Department of Track and Field, Wroclaw University of Health and Sport Sciences, Wroclaw, Poland; ^2^Department of Human Motor Skills, Wroclaw University of Health and Sport Sciences, Wroclaw, Poland; ^3^Department of Sport Team Games, Wroclaw University of Health and Sport Sciences, Wroclaw, Poland; ^4^Department of Physiology, Wroclaw University of Health and Sport Sciences, Wroclaw, Poland; ^5^Department of Physiology and Pathophysiology, Wroclaw Medical University, Wroclaw, Poland; ^6^Department of Physical Education and Health in Biala Podlaska, Faculty in Biala Podlaska, The Jozef Pilsudski University of Physical Education in Warsaw, Biala Podlaska, Poland; ^7^Department of Heart Diseases, Wroclaw Medical University, Wroclaw, Poland; ^8^Center for Heart Diseases, University Hospital in Wroclaw, Wroclaw, Poland

**Keywords:** sport, marathon, muscle stiffness, running economy, endurance performance, older-age runners

## Abstract

**Introduction:**

The research examines the relationship between marathon performance and muscle stiffness changes from pre to marathon in recreational runners aged 50+ years.

**Methods:**

Thirty-one male long-distance runners aged 50–73 years participated in the experiment. The muscle stiffness of quadriceps and calves was measured in two independent sessions: the day before the marathon and 30 min after the completed marathon run using a Myoton device.

**Results and Discussion:**

The 42.195-km run was completed in 4.30,05 h ± 35.12 min, which indicates an intensity of 79.3% ± 7.1% of HRmax. The long-term, low-intensity running exercise (marathon) in older recreational runners and the low level of HRmax and VO2max showed no statistically significant changes in muscle stiffness (quadriceps and calves). There was reduced muscle stiffness (*p* = 0.016), but only in the triceps of the calf in the dominant (left) leg. Moreover, to optimally evaluate the marathon and adequately prepare for the performance training program, we need to consider the direct and indirect analyses of the running economy, running technique, and HRmax and VO2max variables. These variables significantly affect marathon exercise.

## 1. Introduction

Preparing long-distance runners, especially recreational runners and those over 50, to participate in marathons (Ahmadyar et al., 2015) requires a rational strategy of training (Laursen and Jenkins, 2002). This mainly applies to developing the runner’s motor abilities, technical skills, and probably the two most important actions: tactical skills and adequate dietary supplementation during the marathon itself (Keogh et al., 2019; Chmura et al., 2020; Doherty et al., 2020). Tactical skills refer to the o proper distribution of the body’s physical capabilities over a distance. Modern long-distance training has to allow runners to sustain specific loads of long duration. Therefore, continuous running and increased fatigue may cause a runner to experience physiological changes that either enhance or diminish their performance or make it impossible to continue the run (Dotan et al., 1983; Alvero-Cruz et al., 2020; Keogh et al., 2020). Adequate training planning for marathons involves selecting appropriate training methods, maintaining a rational relationship between training loads, competition loads, good recovery, and proper pre-and post-workout supplementation (Hansen et al., 2014).

Most marathon long-distance training programs, especially master runners, are based on regular long, mainly in the aerobic area, runs between 20 and 40 km (Quinn et al., 2011; Casado et al., 2021). The primary purpose of such training is to develop and maintain maximum aerobic power, which is the main requirement to complete a marathon, regardless of the competitors’ level of performance or age. In addition, according to Angus (2014), long-distance runs are intended to enhance running economy (RE). This teaches the athlete to run at a pace (Angus, 2014) as efficiently as possible and translates into actual running pace during the competition (Haney and Mercer, 2011; Kipp et al., 2019). Several researchers (Sproule, 1998; Kyröläinen et al., 2000; Midgley et al., 2007) claim that running economy (RE) is an aerobic demand for the maintenance of running and is referred to as the steady-state oxygen uptake (VO2) related to that speed (Quinn et al., 2011). It is known from practice that after such a long run, the athletes experience considerable muscle damage and soreness (Kyröläinen et al., 2000), which may adversely affect their muscle overload in the next training session (Berg et al., 1998).

Long-distance running competitions are associated with high mechanical stress due to damage to various muscle fibers, metabolic disorders, muscle fatigue (Joyner and Coyle, 2008), and change in muscle stiffness and elasticity (Zierath and Hawley, 2004). From the biomechanical standpoint muscle stiffness is a response to an emitted stimulus, which results from muscle resistance to mechanical lengthening (Rack and Westbury, 1969). According to Wilson et al. (1991) optimal muscle stiffness is significantly correlated with augmentation of muscle training loads.

Increasing muscle stiffness impairs muscle function and, as a consequence, reduces the body’s ability to continue exercising. From a physiological point of view, muscle stiffness is strongly dependent on the size and architecture of the muscles (Brazier et al., 2014; Luu et al., 2015; Behrens et al., 2016) and their specific structural functionality (Zierath and Hawley, 2004). The physiological cross-sectional area (PCSA) was identified as one of the essential features determining muscle stiffness. Other determinants of stiffness are the type of muscle fibers and the percentage of fast-twitch and slow-twitch fibers, as the number and composition of threads, which determine the onset of fatigue and, indirectly, post-training stiffness (Seymore et al., 2017). Multiple training variables can affect muscle stiffness, including the type of muscle work performed, the muscle’s functionality (flexors vs. extensors), and the amount of effort taken until recovery. There are no data on the level of muscle stiffness after prolonged exercise, especially running. It is known that in short-term, dynamic training, more significant muscle damage causes powerful eccentric contractions. Large eccentric muscle contractions during plyometric training cause more significant muscle damage than concentric ones (Kim and Lee, 2015; Wertheimer et al., 2018). This causes more delayed-onset soreness in muscles (Hody et al., 2013; Kanda et al., 2013).

While the relationship between physiological or anthropometric variables and final marathon time has been widely investigated, no study has evaluated muscle stiffness’s relative contribution to marathon performance. Therefore, despite the dozen marathon investigations, there is a lack of clarity about the specific determinants of muscle stiffness on marathon performance. Thus the recipe for success – completing the marathon – remains somewhat elusive. When combined with other performance indicators previously analyzed, assessing muscle analysis would benefit runners and coaches looking to improve their marathon performance. The currently available research devices (MYOTON PRO) are so mobile and it makes reliable measurements that they can be used before and after each running activity in field conditions. Therefore, this study aimed to evaluate marathon performance and evaluate the influence of this long-term running endurance exercise on the changes in muscle stiffness in 50+ marathon runners. Therefore, this study aimed to evaluate marathon performance and evaluate the influence of this long-term running endurance exercise on the changes in muscle stiffness in middle-aged marathon runners. We hypothesize that muscle stiffness will increase with the time that the marathon lasts, no matter what level of training the runner has at the moment.

## 2. Materials and methods

### 2.1. Study design

The main objective of this study was to examine the relationship between marathon performance and changes in muscles stiffness from pre-to post-marathon in recreational runners aged 50+ years. The muscle stiffness of the quadriceps was measured in two independent sessions: the day before the marathon and 30 min after the completed marathon. Myoton measurements of each muscle group (12 points) were taken separately for the left and right legs.

### 2.2. Participants

Thirty-one male long-distance runners aged 50–73 years participated in the experiment. Runners estimated their training experience as 10.61 ± 8.81 years on average. The average result of the marathon run for the study group was 4.30,05 h ± 35.12 min. All participants were free from acute illness or chronic disease and did not take regular medication. The main division criterion was that the runners were over 50 years of age and had participated in at least two marathons in the previous three years. An additional measure was that all participants were actively training for long-distance running for at least one year. Each runner tested signed consent to voluntary participation in the research. Before signing informed consent forms, the participants were informed about the experiment’s aim and the risk of injury. The study protocol was approved by the local Institutional Ethics Committee (Permission 36/2019 AWF Wroclaw). The research was conducted by the Declaration of Helsinki.

### 2.3. Marathon performance

The 37^th^ PKO Wrocław Marathon (Wrocław, Poland, 19 September 2019) was organized by the City of Wrocław, Poland. Since the beginning of the run, The PKO Wrocław Marathon has been organized by the city of Wroclaw and is considered one of Poland’s most significant running events. The PKO Wroclaw Marathon takes place annually at the beginning of September. It was sunny day of the marathon, the air temperature during the start was 21 degrees Celsius, humidity 72%, with a wind of 3.3 m/s. At the end of the run, the temperature rose to 24 degrees Celsius, humidity 46%, with a wind of 5 m/s. The data on the running time was obtained from the electronic database of the marathon’s organizers. It included the number of runners who started and finished the run, the personal identification number of the run, and the place and time of the run for each participant of the marathon. The individual runtime registered in the event was automatically measured using a radio frequency identification chip system. Intermediate times every 5 km were measured for the experimental group to analyze their running pace variability accurately. In addition, the heart rate (HR) was recorded using a monitor (Polar RS300X GPS; Finland) to examine each participant during the marathon run.

### 2.4. Study protocol

#### 2.4.1. Applied equipment

The Myoton PRO (Myoton AS, Estonia and Myoton Ltd., London) is a wireless hand-held device placed perpendicular to the skin over the muscle being measured. This device was applied under constant preload (0.18 N) to pre-compress subcutaneous tissues and exert a brief (15 ms) mechanical tap at a predetermined force (0.4 N), followed by a quick release, thereby causing dampened oscillations that are recorded by the testing probe.^1^ The non-neural tone or tension was calculated from the signal spectrum Fmax [fast Fourier transform (FFT)] and had the frequency (Hz) of the dampened oscillations. Stiffness (N/m) was characterized by the muscle’s ability to resist an external force that modified its shape (Pisano et al., 2000). Elasticity was measured by the logarithmic decrement (log) of the dampened oscillations (dissipation of mechanical energy during one oscillation cycle), thus reflecting the ability of the tissue to recover its shape after being deformed (Mullix et al., 2012).

#### 2.4.2. Muscle stiffness measurements

##### 2.4.2.1. Field experiments

The first muscle stiffness sample was collected on the day before the marathon. The quadriceps muscle of the thigh and the triceps muscle of the calf were measured. All measurements were performed in a designated room. Rigidity measurements were taken at rest the day before the marathon. Subsequent measurements were made 1–2 h before the start and just after the end of the marathon run. All tests were performed by the same trained person to operate the MYOTON device.

**Figure 1:**
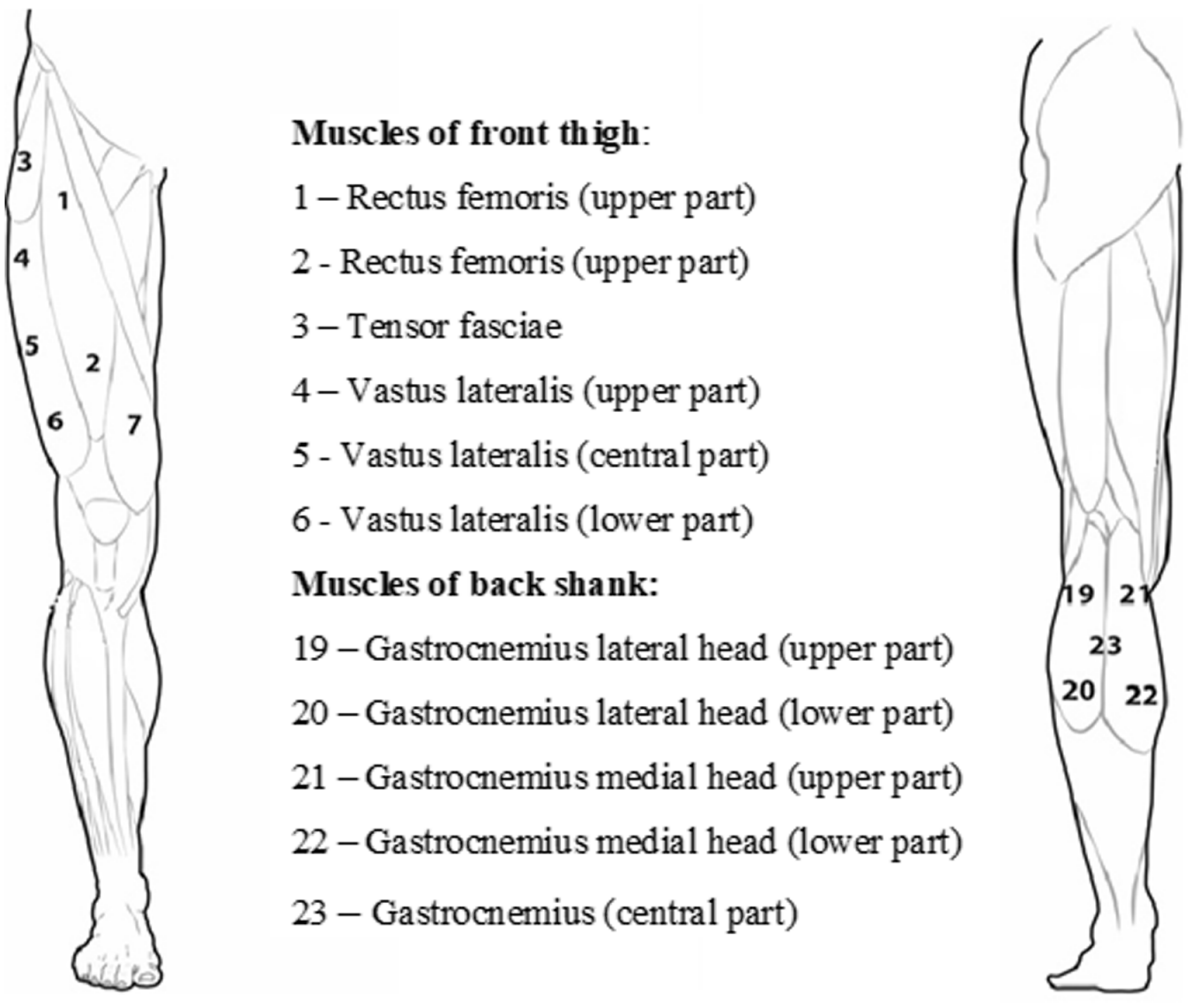
Arrangement of the measurement points of the quadriceps muscle of the thigh and the triceps muscle of the calf.

The participants were prone on their backs or their stomachs on a unique bed, and they rested for 10 min before muscle stiffness measurements were taken. Testing sites on each muscle were located using a tape measure and marked using a skin-safe pen (Figure 1). A pillow was placed under the head, and a unique roller pillow was placed under the lower leg to aid relaxation. One series of three single Myoton measurements of each muscle group (12 points) were measured separately for the left and right legs. In addition, for a better understanding of the problem, the functionality of the lower limb was also determined - the dominant and non-dominant leg. The dominant leg for a particular runner was determined based on the information provided by the marathoner in the questionnaire.

The reliability between trials (within session) of the one selected muscles (two series of 10 single measurements) of each group was tested using intraclass correlation coefficient (ICC) model. Domholdt (1993) classification scales for interpreting ICCs was used: very high =1.00–0.90; high = 0.89–0.70; moderate = 0.69–0.50; low = 0.49–0.26. This indicated that Rectus femoris reach (ICC = 0.82) and Gastrocnemius (ICC = 0.85). The high reliability coefficient indicated that applied tests represent consistent measurement of muscle stiffness data among the runners.

##### 2.4.2.2. Resting recordings

The second sample was collected immediately after the marathon run. All measurements were performed in a tent near the marathon finish line. Testing sites on each muscle belly were identified using a tape measure and marked using a skin-safe pen. A pillow was placed under the head, and a unique roller pillow was placed under the lower leg to aid relaxation. Again, a series of three single Myoton measurements of each muscle group (12 points) was taken separately for the left and right legs.

#### 2.4.3. Maximal oxygen consumption measurement

All subjects underwent a maximal cardiopulmonary exercise test on a motorized treadmill (Trackmaster TMX425, Full Vision, Inc., KS, United States). After a 5-min running at 8 km/h (warm-up), the protocol started, and the treadmill speed was increased by 1 km/h every 2 min, in a stepwise fashion. The treadmill inclination was kept constant at 1°. The protocol was continued until exhaustion. Oxygen uptake (VO2, mL/min/kg) and instantaneous minute ventilation (VE, L/min) were measured breath by breath (Cosmed Quark CPET, Rome, Italy) and averaged every 30s. The highest values of VO2 and VE were taken as VO2max (mL/kg/min) and maximal minute ventilation (VEmax, l/min), respectively.

### 2.5. Statistical analysis

Data were tested for normality using the Shapiro–Wilk test and homogeneity of variance (Levene’s test). Descriptive statistics included the mean, SD, and SE. To compare the mean values of the examined variables, repeated measures of one-way ANOVA were used. The independent variable is the time needed to complete the marathon run, whereas the dependent variables were muscle stiffness (MFT, MBS, and HR). After a significant main or interaction effect was established, the data were evaluated with a post-hoc Fisher’s LSD test. The level of statistical significance was set at *p* = 0.05. Additionally, Cohen’s *d* was calculated, and the effect sizes were determined: 0.35 for small effect size, 0.35 and 0.65 for medium effect size, and 0.65 for large effect size (Cohen, 1988). The relationship between the variables was determined using Pearson’s product–moment correlation. Statistical power was set to be >0.90 at *p* = 0.05. All statistical analyses were made using the STATISTICA ver. 13.1 (StatSoft. Inc., United States) software package.

Reliability between trials (within-session) for one of the selected muscles (two series of 10 single measurements) of each group was tested using the intraclass correlation coefficient (ICC) model. Domholdt classification scales (Carter et al., 2011) for interpreting ICCs were used: very high = 1.00–0.90; high = 0.89–0.70; moderate = 0.69–0.50; and low = 0.49–0.26. ICCs were found for the rectus femoris (ICC, 0.82), biceps femoris (ICC, 0.86), tibialis anterior (ICC, 0.91), and gastrocnemius (ICC, 0.85). The high reliability of the coefficients indicated that the tests resulted in inconsistent measurements of muscle stiffness among the Marathon runners.

## 3. Results

The 42.195-km run was completed in 4.30,05 h ± 35.12, which indicates an intensity of 79.3% ± 7.1% of HRmax. The average body height of the marathon runners was 175.61 ± 5.74 cm, their body weight was 76.17 ± 7.73 kg, and their BMI was 24.44 ± 2.32. A low level of HRmax and VO2max was visible. Similar relationships can be seen in the case of VO2 at the aerobic threshold (VT1) and anaerobic threshold (VT2). The participant’s percentage of the Wrocław Marathon on VT1 achieved 76.23% VO2max. The possibilities at the VT2 hall were 91.3 and 84.65% HRmax (Table 1).

**Table 1:** Baseline characteristics of the participants, presented as mean ± SD.

Variables	Mean ± SD
Age (years)	57.32 ± 6.25
Body weight (kg)	75.36 ± 7.89
Height (cm)	175.61 ± 5.74
Body mass index (kg/m^2^)	24.44 ± 2.32
Training experience	10.61 ± 8.81
HR max	169 ± 15.71
VO_2_max (mL/min/kg)	44.51 ± 3.63
VO_2_ VT1 (mL/min/kg)	33.93 ± 4.28
fR-breaths/min (VT1)	35.81 ± 6.16
VE (VT1) L/min	76.95 ± 15.32
HR (VT1)	142.10 ± 17.89
VO2 VT2 (mL/min/kg)	40.64 ± 4.28
fR-breaths/min (VT2)	45.20 ± 5.75
VE (VT2) L/min	110.12 ± 17.55
HR (VT2)	161.50 ± 16.19

**Figure 2:**
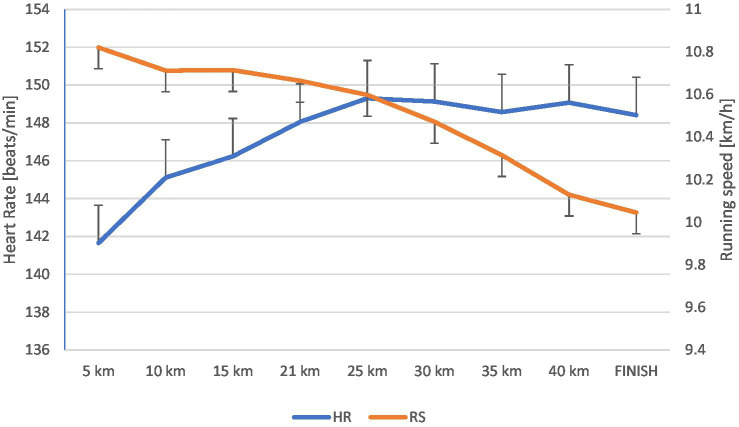
Changes in heart rate [beats/min] and running speed [km/h] during the marathon (mean ± SD).

Figure 2 shows the speed variability with the division into individual sections (every 5 km) and the average HR on these sections. It can be seen that the beginning of the drop in speed starts at 12 km and continues to the end of the run. Along with the decrease in rate, there was a gradual increase in HRmax, which lasted until the marathon’s end.

The analysis of muscle stiffness levels in relation to their subsequent measurements (before and after the marathon) revealed that a significant effect was observed only for the left calf (*F* = 6.534(1); *p* = 0.016; medium effect size). No significant effect was found for the left quadriceps (*F* = 2.393(1); *p* = 0.132), the right calf (*F* = 3.493(1); *p* = 0.071), or the right quadriceps (*F* = 0.876(1); *p* = 0.357; Table 2).

**Table 2:** Differences in muscle stiffness before and after the marathon (mean ± SD).

Leg	Muscle group	Before marathon	After marathon	*F*	*p*	Cohen’s *d*
Left	Calf	302.88 ± 33.14	292.10 ± 22.88	6.534	0.016	0.38
	Quadriceps	310.43 ± 29.46	301.92 ± 38.00	2.393	0.132	0.25
Right	Calf	313.19 ± 41.86	303.39 ± 28.29	3.493	0.071	0.28
	Quadriceps	294.94 ± 20.54	297.94 ± 25.59	0.876	0.357	0.13

The Spearman’s rank correlation analysis did not reveal any significant correlation between running speed or HR level, measured over the entire distance divided into 5 km sections, and muscle stiffness of the quadriceps and triceps calf muscles. On the other hand, significant relationships occurred only between the VO2max measurement (measured before the race) and the speed at each of the 5-km sections of the marathon distance and the finish (*p* = 0.000034 and *p* = 0.000239, respectively).

## 4. Discussion

This research examines the relationship between marathon performance and changes in muscle stiffness from pre to marathon in middle-aged recreational marathon runners. The hypothesis regarding muscle stiffness was not supported, as the current investigation revealed significantly lower levels of stiffness post-marathon for the calf muscles in the left leg (*p* = 0.016). No significant changes were noted in muscle stiffness at the post-marathon assessment in the other two tested muscle groups (quadriceps, left and right lower limbs, and calf in the right stem).

The explanation of this phenomenon is likely to be difficult because none of the previously described studies have documented the impact of a prolonged running effort, commonly defined as a marathon, on muscle stiffness. Additionally, this requires the consideration of indirect analyses of other variables which affect the marathon effort. This approach is also considered difficult because many of the factors to be analyzed were not included in this experiment. This is because many of these variables are difficult to measure without interfering with the running autonomy. However, it has been well described how long-distance running directly impacts the running economy (RE) (Quinn et al., 2011). It seems reasonable to combine all these factors, due to the non-exclusive relationships, to optimally assess the marathon effort and its direct impact on changes in the runner’s body after such a long effort. Knowing this may help runners improve their marathon performance and develop an appropriate training program, optimally preparing them to run 42.195 m, regardless of their championship, intermediate, or recreational level. An essential element of such an analysis is the division into sex, mainly into age categories, with a particular emphasis on 50+ (Beck et al., 2016).

It is well known that marathon performance depends on the running economy (RE) in all the world’s weariness. RE is an ‘aerobic demand’ to maintain a reasonable pace: speed over distance. It is defined as the stationary oxygen uptake (VO2) associated with this speed (Sproule, 1998; Kyröläinen et al., 2000). Comparing our participants with younger marathon runners aged 43.9 ± 8.3, the values of HRmax were lower by an average of 9.2 (bpm) and 4.29 (mL/min/kg; Nikolaidis and Knechtle, 2018). More significant differences occur compared to the group of recreational runners (63 ± 32 km/week) aged 34 ± 8 years. The differences in HRmax and VO2max are 14.5 (bpm) and VO2max by 18.69 (mL/min/kg), respectively, favoring the younger runners (Lanferdini et al., 2020). Similar relationships can be seen in the case of VO2 at the aerobic threshold (VT1) and anaerobic threshold (VT2). Younger runners are characterized by higher VO2 at these thresholds (3.67 for VT1 and 12.86 for VT2; Lanferdini et al., 2020). Despite the lower values of these indicators, the marathon runners studied in Wrocław consumed more oxygen about their abilities than younger recreational athletes. The percentage of participants of the Wrocław Marathon on VT1 achieved 76.23% VO2max, while the competitors studied by Lanferdini et al. (2020) only 59.49% VO2max. Comparing the capabilities at the VT2 threshold, the results were 91.3 and 84.65% HRmax, respectively. Despite this, our marathon runners showed a strong relationship between Vo2 max and speed on each subsequent 5-km section (from 0.000034 to 0.000239). This previous confirmed research found a strong relationship between VO2 max and the level of effort in a marathon run.

It is also evident that the RE must be associated with the marathon runner’s running technique and that this, in turn, depends on the resistance of the runner’s body to fatigue and falling running speed. In our experiment, marathon runners began to experience a drop in running speed after 12 km of a race. A continuous, slow decrease in rate started from that moment on, which amounted to approximate 5.6% at the finish line. This did not confirm the reports of Hettinga et al. (2019) that during the late stages of the marathon (the last 10–15 km), a considerable deceleration usually occurs. This affects even world-class runners and is recognized by runners as ‘hitting the wall’ (Buman et al., 2009). This is probably because our runners are classified as slow, recreational runners, over 50 years of age, so their marathon effort can be defined as prolonged (4.30,05 h ± 35.12 min.) but of low intensity.

On the other hand, world-class marathon runners have developed training strategies to manage or prevent fatigue and sharp drops in running speed (Hanley et al., 2020). The studies by Buckalew et al. (1985) and Chan-Roper et al. (2012) regarding the effects of fatigue on running technique showed that technique changed by decreases in step length rather than step frequency. These changes were directly responsible for the decreased speed. Marathon runners are predominantly rear-foot strikers, valid for world-class (Hanley et al., 2019) and recreational long-distance runners (Larson et al., 2011). This can be applied to our marathon runners with an indication of the activity of the left leg and with particular emphasis on the triceps muscle of the calf. They noticed a few disadvantages in this matter. A significant potential biomechanical limitation of landing with a rear-foot strike pattern is that the foot lands in front of the whole body’s center of mass. This increases the braking force and directly impacts the speed, mainly reducing it by the resulting weaker take-off. This negatively influences the step length by shortening it. The second disadvantage of the running technique when fatigue appears is that landing almost the whole foot on the ground during the early stance and continuing during the main amortization phase significantly increases contact time. In turn, the high center of mass is achieved through knee flexion. The greater the knee flexion, the longer the foot–ground contact time, and the higher the speed reduction. Additionally, according to Derrick et al. (2002), fatigue may decrease the utilization of the stretch-shortening mechanism, especially in the hip and knee joints. This causes the knee flexors and extensors to tire more quickly, which results in reduced leg stiffness. Despite this assumption, the relationship between running speed on each 5-km stretch (increasing fatigue with each km) and muscle stiffness was not confirmed with no change in muscle stiffness. However, a much more significant correlation was found for the triceps muscle of the calf (mean significance level: *p* = 0.354612). This can be confirmed because this muscle has a more significant functional impact on the running step technique. It weakens the ground reaction forces, thus significantly extending the contact time (Mercer et al., 2002). These elements mean a considerable speed reduction, and the runners thus achieve poor results. In addition, these undesirable factors should be eliminated in training to achieve optimal results in the marathon about motor preparation. At the same time, these parameters, which should not weaken the running technique, had a positive effect on muscle stiffness. This did not change after the marathon effort compared to the measurements before the race.

Changes in the mechanical properties of the muscles observed after prolonged physical activity may be associated with increased joint stiffness. In terms of performance, the increased stiffness was associated with increased speed, increased jump velocity, jump height, and running economy (measured by oxygen consumption; Sadeghi et al., 2018). According to Beckett et al. (2017), followed by Kerdok et al. (2002), a critical determinant of running economy is the spring-like storage and return of elastic energy from the leg during a stance. Here we have to distinguish between muscle stiffness and joint stiffness, often equated with leg spring stiffness. The latter measures the stiffness of the muscle and tendon, but regarding how well a runner can recoil the elastic energy generated during ground contact in each stride. Therefore, increased joint stiffness, mainly by eccentric contraction movement, shorten ground contact (Kerdok et al., 2002; Bus, 2003) and generate more elastic energy. This indicates an improvement in running economy over time and an increase in delayed-onset muscle soreness.

According to Beckett et al. (2017), it can be concluded that the assessment of older runners may be indirectly based on leg stiffness, through reduced tendon stiffness (Karamanidis et al., 2005; Magnusson et al., 2008), lower active peak ground vertical reaction forces (GRF) (Bus, 2003), and greater flexion at the knee joint at landing (Fukuchi and Duarte, 2008; Kulmala et al., 2014). This suggests that leg spring stiffness decreases with age (Beck et al., 2016). Did this occur in our marathon runners?

One of the limitations of this study is the absence of subsequent measurements of muscle stiffness, e.g., 12 h or 24 h after completing the marathon. This was not due to the technical feasibility of the measures but to the personal reasons of the competitors. Such measurements would have also allowed us to observe DOMS changes about the delayed changes. Another limitation is the lack of a running technique evaluation on video recording, e.g., 15 km or 40 km into the race. This would have allowed us to correctly describe the marathoners’ running technique and juxtapose it with VO2 to evaluate their running economy.

## 5. Conclusion

The long-term, low-intensity running effort (marathon) in older recreational runners, along with a low HRmax and VO2max, showed no statistically significant changes in muscle stiffness (quadriceps and calf muscles). There was, however, reduced muscle stiffness, but only in the triceps calf of the dominant (left) leg. Additionally, when we consider the failure to keep an optimal running economy, expressed as a technical disorder (shortened running step, increased ground contact time, lowering the legs in the knee joint) and increasing fatigue, we can surmise why muscle stiffness did not change post-exercise. Therefore, this experiment did not confirm the hypothesis that mechanical muscle properties and resting tone may change after prolonged exercise.

From a practical point of view, the lack of changes in muscle stiffness in the post-marathon suggests that the training of the marathon mentioned above runners are based on too low intensities. It is closely related to the results they achieved in the experiment. Therefore, the improvement of the development in the marathon, especially in the advanced age of marathon runners, will occur by increasing the running training with greater intensity. This will allow the runner to experience increased muscle stiffness during training. Then transfer it to the competition.

## Data availability statement

The raw data supporting the conclusions of this article will be made available by the authors, without undue reservation.

## Ethics statement

The studies involving human participants were reviewed and approved by the local Institutional Ethics Committee (Senacka Komisja ds. Badań Naukowych przy Akademii Wychowania Fizycznego we Wrocławiu). The patients/participants provided their written informed consent to participate in this study.

## Author contributions

KM, JC, DM, DP, and PP conceived and designed the experiments. KM, JC, DM, DP, PC, and MK performed the experiments. KM, JC, DM, DP, PC, and MK analyzed the data. BeP, RS, MW, AN-C, WŁ, DA, SW, PP, and BaP interpreted the results. KM and DM drafted and edited manuscript. All authors critically revised paper and approved the final version of the manuscript. All authors contributed to the article and approved the submitted version.

## Funding

This research was financially supported by the Ministry of Science and Higher Education (Poland)/Wroclaw University of Health and Sport Sciences. The funders had no role in study design, data collection and analysis, decision to publish, or preparation of the manuscript.

## Conflict of interest

The authors declare that the research was conducted in the absence of any commercial or financial relationships that could be construed as a potential conflict of interest.

## Publisher’s note

All claims expressed in this article are solely those of the authors and do not necessarily represent those of their affiliated organizations, or those of the publisher, the editors and the reviewers. Any product that may be evaluated in this article, or claim that may be made by its manufacturer, is not guaranteed or endorsed by the publisher.

## Footnotes

^1^ http://www.myoton.com/en/Technology/Technical-specification
